# Supplementary material for: Modelling students’ cognitive achievement skills using the alpha power transformed Lindley probability distribution
Source: PLoS One. 2025 Jul 24;20(7):e0328942. doi: 10.1371/journal.pone.0328942 (PMC12289089; doi:10.1371/journal.pone.0328942)
Supplement: S1 Appendix — (DOCX) [file pone.0328942.s001.docx]

# **Supporting information**

# **S1 Appendix. R code.**

loglik_APTL_regression <- function(params, y, X) {

# Extract parameters

alpha <- exp(params[1]) + 1 # Ensure alpha > 1

beta <- exp(params[2]) # Ensure beta > 0

delta <- exp(params[3]) # Ensure delta > 0

theta <- exp(params[4]) # Ensure theta > 0

cat("alpha:", alpha, "beta:", beta, "delta:", delta, "theta:", theta, "\n")

# Extract regression coefficients for fixed effects

beta_coeff <- params[5:(4 + ncol(X))]

# Linear predictor (fixed effect part)

linear_pred <- X %*% beta_coeff

cat("linear_pred:\n", linear_pred, "\n")

# Calculate rate parameter (adjusted by the linear predictor)

rate <- theta * exp(linear_pred)

cat("rate:\n", rate, "\n")

# Check for invalid or NA rate values

if (any(is.na(rate)) || any(rate <= 0)) {

cat("Invalid or NA values detected in rate. Returning Inf.\n")

return(Inf)}

# Calculate the PDF (same form as before)

pow <- -((theta * delta + beta + theta * beta * y) * exp(-theta * y)) / (theta * delta + beta)

pdf <- (log(alpha) * alpha * (theta^2) * (delta + beta * y) * exp(-theta * y) * alpha^(pow)) /

((alpha - 1) * (theta * delta + beta))

cat("pdf:\n", pdf, "\n")

# Check for invalid PDF values

if (any(is.na(pdf)) || any(pdf <= 0)) {

cat("Invalid or NA values detected in PDF. Returning Inf.\n")

return(Inf)}

# Calculate total log-likelihood

total_log_likelihood <- sum(log(pdf))

cat("total_log_likelihood:", total_log_likelihood, "\n")

# Check for finite log-likelihood

if (!is.finite(total_log_likelihood)) {

cat("Total log-likelihood is not finite. Returning Inf.\n")

return(Inf)}

return(-total_log_likelihood) # Return negative for minimization}

#Model Fitting

#Since we removed the random effects, we also need to update the

#parameter initialization and fitting process.

# Prepare data

# Convert categorical variables to factors

categorical_vars <- c("FEducation", "Elec", "Transportation", "MEducation", "Residence", "Region")

for (var in categorical_vars) {

paper3[[var]] <- as.factor(paper3[[var]])}

# Define response variable

y <- paper3$MathScore

# Define covariates and scale them

X <- model.matrix(~ Residence + Transportation + HouseSize + Elec + Region+MEducation + FEducation, data = paper3)

# Initialize parameters

initial_params_regression <- c(

log(2), # log(alpha - 1)

log(1.5), # log(beta)

log(1.5), # log(delta)

log(1.5), # log(theta)

rep(0, ncol(X)) # Initial values for the covariates' coefficients)

# Fit the model using optimization

fit_regression <- optim(

par = initial_params_regression,

fn = loglik_APTL_regression,

y = y,

X = X,

method = "Nelder-Mead",

control = list(maxit = 10000, trace = 1, reltol = 1e-6))

# Extract estimated parameters

estimated_params_regression <- fit_regression$par

log_likelihood_regression <- -fit_regression$value # Negative log-likelihood

AIC_regression <- 2 * length(initial_params_regression) - 2 * log_likelihood_regression

BIC_regression <- log(length(y)) * length(initial_params_regression) - 2 * log_likelihood_regression

# Display results

cat("Estimated Parameters:\n", estimated_params_regression, "\n")

cat("Log-Likelihood:", log_likelihood_regression, "\n")

cat("AIC:", AIC_regression, "\n")

cat("BIC:", BIC_regression, "\n")

# Extract coefficients

coefficients <- estimated_params_regression[5:(4 + ncol(X))]

cat("Estimated Coefficients:\n")

print(coefficients)
