# Supplementary material for: Modelling students’ cognitive achievement skills using the alpha power transformed Lindley probability distribution
Source: PLoS One. 2025 Jul 24;20(7):e0328942. doi: 10.1371/journal.pone.0328942 (PMC12289089; doi:10.1371/journal.pone.0328942)
Supplement: Attachment — Submitted filename: PONE-D-25-16740_reviewer-v1.pdf [file pone.0328942.s002.pdf]

Referee Report on

"Modelling Students' Cognitive Achievements in Mathematics Using the Alpha Power Lindley Probability Distribution"

This paper is **good** prepared and organized. It proposes regression modeling using the Alpha Power Transformation Lindley (APTLD) probability distribution for the application of cognitive achievement in mathematics skill scores. The study also compares this distribution with other competitive probability distributions. Young Lives data was used for the present study, and different explanatory variables were included. The results show that the mean cognitive achievement in mathematics skill scores (37.01) is greater than the median (33.33), indicating that the data are positively skewed or right skewed. The comparison of the fitted models suggests APTLD as the best model.

I recommend accepting this paper **after major revisions**:

1. Moderate English changes required.

2. The author should write the motivation of this study clearly.

3. The authors must rewrite the introduction and add the section titles and summary about them.

4. The authors must proof the APTLD follows an exponential family before start doing the GLM.

5. The authors must include the *direct link* for the dataset used in this study.

6. In probability, we always use capital letters to denote the random variables. Also, the estimated parameter with add a cap up of it, for example $\hat{\beta}$. So, the authors must make this change

7. η_i is equation (2) should be changed to $X_i\beta$.

8. I recommend adding the R codes to the appendix.
