# Supplementary material for: Modelling students’ cognitive achievement skills using the alpha power transformed Lindley probability distribution
Source: PLoS One. 2025 Jul 24;20(7):e0328942. doi: 10.1371/journal.pone.0328942 (PMC12289089; doi:10.1371/journal.pone.0328942)
Supplement: Attachment — Submitted filename: PONE-D-25-16740_reviewer-v2.pdf [file pone.0328942.s004.pdf]

Referee Report on

"Modelling Students' Cognitive Achievements in Mathematics Using the Alpha Power Lindley Probability Distribution"

I recommend accepting this paper **after major revisions**:

1. The authors must rewrite the introduction and add the section titles and summary about them by mentioning that section by section with section number.

2. The authors must prove the APTLD follows an exponential family before starting doing the GLM.

3. I recommend adding the R codes to the appendix (I can't see the appendix in the modified version).
