# Supplementary material for: Modelling students’ cognitive achievement skills using the alpha power transformed Lindley probability distribution
Source: PLoS One. 2025 Jul 24;20(7):e0328942. doi: 10.1371/journal.pone.0328942 (PMC12289089; doi:10.1371/journal.pone.0328942)
Supplement: Attachment — Submitted filename: PONE-D-25-16740_reviewer-v3.pdf [file pone.0328942.s006.pdf]

Referee Report on

“Modelling Students’ Cognitive Achievements in Mathematics Using the Alpha Power Lindley Probability Distribution”

I have checked the revised paper again, now the manuscript has been sufficiently improved to warrant publication in PLOS ONE.
