# Supplementary material for: Modelling students’ cognitive achievement skills using the alpha power transformed Lindley probability distribution
Source: PLoS One. 2025 Jul 24;20(7):e0328942. doi: 10.1371/journal.pone.0328942 (PMC12289089; doi:10.1371/journal.pone.0328942)
Supplement: Attachment — Submitted filename: Response to Reviewers.docx [file pone.0328942.s003.docx]

**Authors responses for the reviews comments May 29, 2025**

| **Reviewers comments** | |
| --- | --- |
| **Reviewer 1 comments to the author** | **Authors’ responses** |
| 1. The authors must state more clearly their original methods, their original results and conclusions, the novelty of their study. | Thank you for your comment and suggestion. We have made a correction in the revised manuscript. |
| 2. Abstract the topic's background; the methods, should have summarized the most important outcomes of their study and the conclusions that one could draw. | We have corrected it in the revised manuscript. |
| 3. The authors should in detail present the findings and their main implications, also highlighting current limitations of their study, and briefly mention some precise directions that they intend to follow in their future research work. | We have included in the updated manuscript. |
| **Reviewer 2 comments to the author** | **Authors’ responses** |
| 1. Moderate English changes required | We have corrected it. |
| 2. The author should write the motivation of this study clearly. | Thank you. We have corrected it. |
| 3. The authors must rewrite the introduction and add the section titles and summary about them. | We have included some important points in the introduction section. |
| 4. The authors must proof the APTLD follows an exponential family before start doing the GLM. | We have corrected it |
| 5. The authors must include the direct link for the dataset used in this study. | We have states the datasets clearly. |
| 6. In probability, we always use capital letters to denote the random variables. Also, the estimated parameter with add a cap up of it, for example β ̂. So, the authors must make this change | Thank you, we have corrected it. |
| 7. η_i is equation (2) should be changed to X_i β. | Thank you, we have corrected it. |
| 8. I recommend adding the R codes to the appendix. | I have included |

Manuscript is revised and attached.
