# Supplementary material for: Modelling students’ cognitive achievement skills using the alpha power transformed Lindley probability distribution
Source: PLoS One. 2025 Jul 24;20(7):e0328942. doi: 10.1371/journal.pone.0328942 (PMC12289089; doi:10.1371/journal.pone.0328942)
Supplement: Attachment — Submitted filename: Response_to_Reviewers_auresp_2.docx [file pone.0328942.s005.docx]

**Authors responses for the reviews comments June 26, 2025**

| **Reviewers comments** | |
| --- | --- |
| **Reviewer 2 comments to the author** | **Authors’ responses** |
| 1. The authors must rewrite the introduction and add the section titles and summary about them by mentioning that section by section with section number. | Thank you for your comment and suggestion. We have made a correction in the revised manuscript. |
| 2. The authors must prove the APTLD follows an exponential family before starting doing the GLM. | We have corrected it in the revised manuscript. |
| 3. I recommend adding the R codes to the appendix (I can’t see the appendix in the modified version). | We have included in the supporting information section. |

Manuscript is revised and attached.
